# 1199. Decreased Human Respiratory Syncytial Virus Activity during the COVID-19 Pandemic in Japan: An Ecological Time-Series Analysis, 2014 through 2020

**DOI:** 10.1093/ofid/ofab466.1391

**Published:** 2021-12-04

**Authors:** Keita Wagatsuma, Iain S Koolhof, Reiko Saito

**Affiliations:** 1 Division of International Health (Public Health), Graduate School of Medical and Dental Sciences, Niigata University, Niigata, Japan, Niigata, Niigata, Japan; 2 College of Health and Medicine, School of Medicine, University of Tasmania, Hobart, Australia, Hobart, Tasmania, Australia

## Abstract

**Background:**

Non-pharmaceutical interventions (NPIs), such as sanitary measures and travel restrictions, aimed at controlling the severe acute respiratory syndrome coronavirus 2 (SARS-CoV-2), may affect the transmission dynamics of human respiratory syncytial virus (HRSV). We aimed to quantify the contribution of the sales of hand hygiene products and the number of international and domestic airline passenger arrivals on HRSV epidemic in Japan.

**Methods:**

The monthly number of HRSV cases per sentinel site (HRSV activity) in 2020 was compared with the average of the corresponding period in the previous 6 years (from January 2014 to December 2020) using a monthly paired *t*-test. A generalized linear Poisson regression model was used to regress the time-series of the monthly HRSV activity against NPI indicators, including sale of hand hygiene products and the number of domestic and international airline passengers, while controlling for meteorological conditions (monthly average temperature and relative humidity) and seasonal variations between years (2014–2020).

**Results:**

The average number of monthly HRSV case notifications in 2020 decreased by approximately 85% (*P* < 0.001) compared to those in the preceding 6 years (2014–2019) (Figure 1A). For every average ¥1 billion (approximately &9,000,000/£6,800,00) spent on hand hygiene products during the current month and 1 month before (lag 0-1 months) there was a 0.22% (*P* = 0.02) decrease in HRSV infections (Table 1). An increase of average 1,000 domestic and international airline passenger arrivals during the previous 1–2 months (lag 1–2 months) was associated with a 4.6×10^−4^% (*P* < 0.001) and 1.1×10^−3^% (*P* = 0.007) increase in the monthly number of HRSV infections, respectively.

Figure 1. Monthly seasonal variations of number of HRSV activity, NPI indicators, and meteorological conditions during 2014-2020.

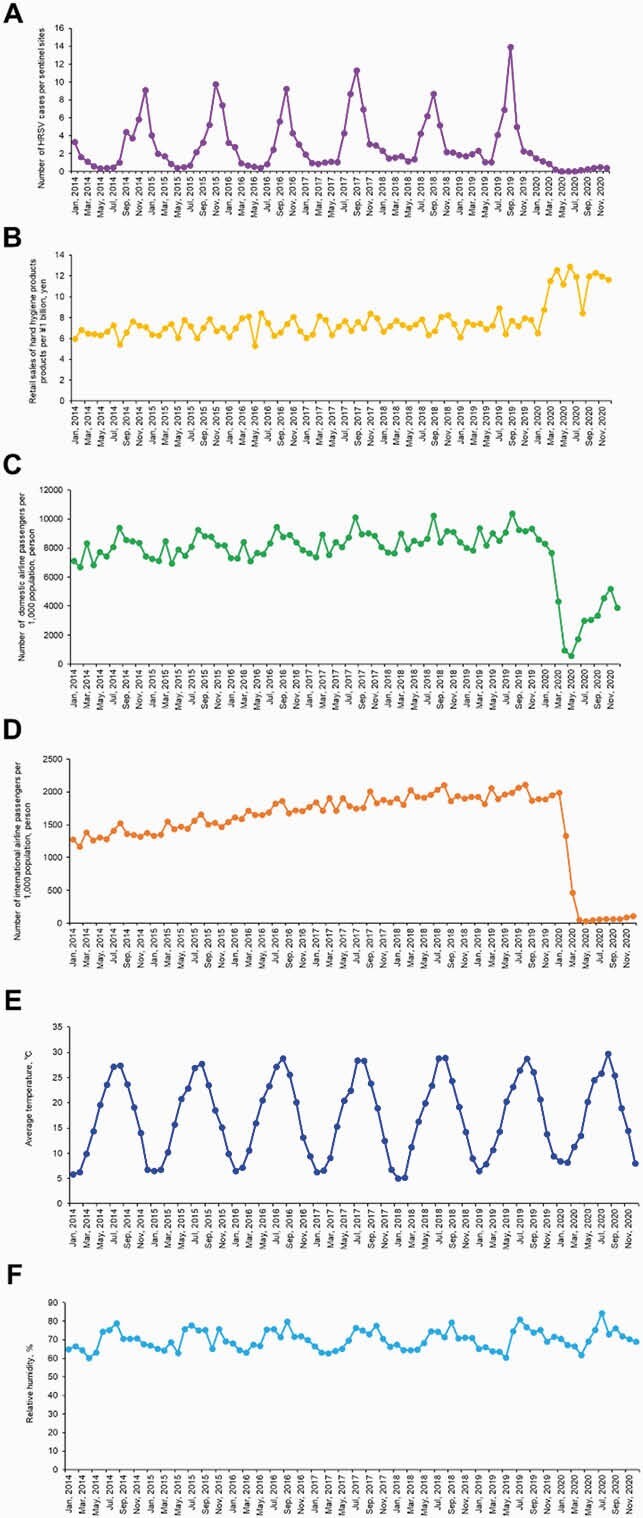

(A) Monthly seasonal variations of number of HRSV cases per sentinel sites based on national HRSV surveillance data during 2014-2020. (B) Monthly seasonal variations of retail sales of hand hygiene products per ¥1 billion (unit: yen) during 2014-2020. (C) Monthly seasonal variations of number of domestic airline passengers per 1,000 population (unit: person) during 2014-2020. (D) Monthly seasonal variations of number of international airline passengers per 1,000 population (unit: person) during 2014-2020. (E) Monthly seasonal variations of average temperature (unit: ℃) throughout Japan during 2014-2020. (F) Monthly seasonal variations of relative humidity (unit: %) throughout Japan during 2014-2020.

Table 1. Generalized linear Poisson regression model for the monthly number of human respiratory syncytial virus cases among prefectures in Japan.

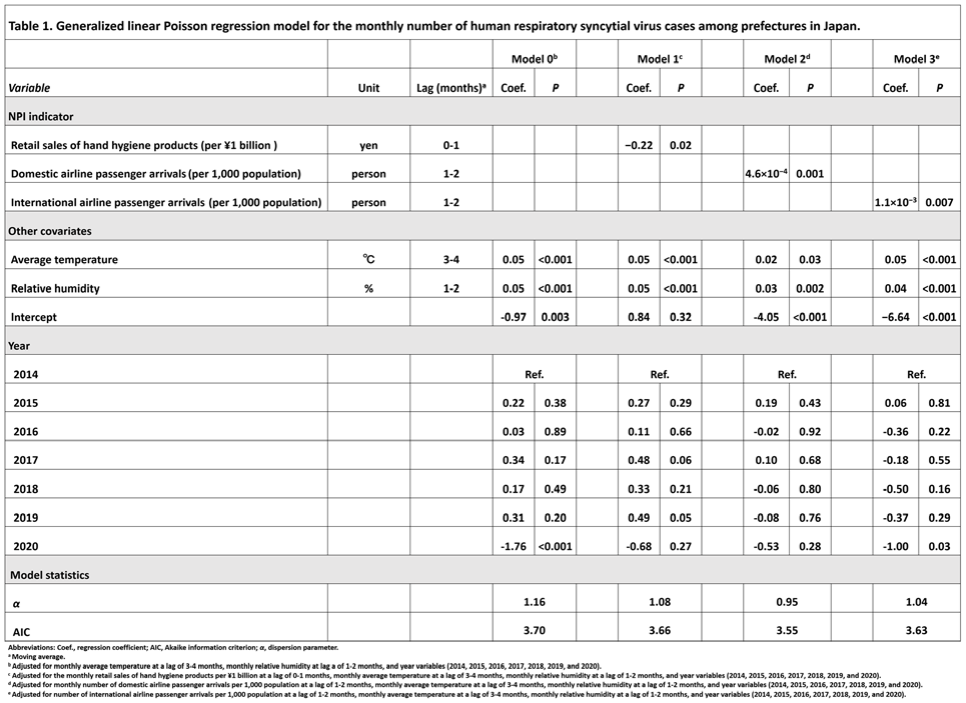

**Conclusion:**

This study suggests that there is an association between the decrease in the monthly number of HRSV cases and improved hygiene and sanitary measures and travel restrictions for COVID-19 in Japan, indicating that these public health interventions can contribute to the suppression of HRSV activity. These findings may help in public health policy and decision making.

**Disclosures:**

**All Authors**: No reported disclosures

